# Picocavities: a Primer

**DOI:** 10.1021/acs.nanolett.2c01695

**Published:** 2022-07-06

**Authors:** Jeremy J. Baumberg

**Affiliations:** † Nanophotonics Centre, Cavendish Laboratory, University of Cambridge, Cambridge CB3 0HE, United Kingdom

**Keywords:** picocavity, nanocavity, plasmonics, SERS, Raman scattering, adatoms

## Abstract

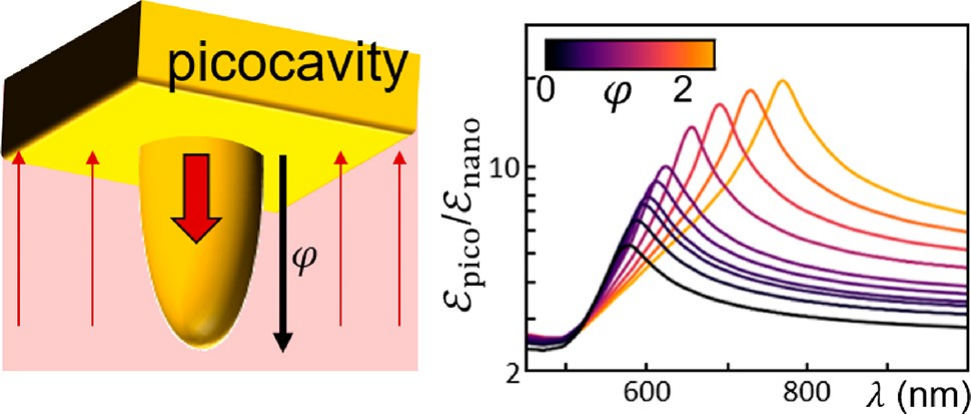

Picocavities are sub-nanometer-scale optical cavities recently found to trap light, which are formed by single-atom defects on metallic facets. Here, we develop simple picocavity models and discuss what is known and unknown about this new domain of atom-scale optics, as well as the challenges for developing comprehensive theories. We provide simple analytic expressions for many of their key properties and discuss a range of applications from molecular electronics to photocatalysis where picocavities are important.

## Introduction to Picocavities

The observation of clear and persistent transient vibrational lines in surface-enhanced Raman spectra (SERS) has opened up a new domain of spectroscopy for single molecules and their interactions with metal surfaces. While fleeting phenomena have been seen since the 1990s, measurements of vibrational pumping in 2016 finally allowed a rigorous evaluation of the optical mode volume,^1^ which was found to below 1 nm^3^. These modes are thus called picocavities and are formed by single atom surface defects known as adatoms.

**Figure 1:**
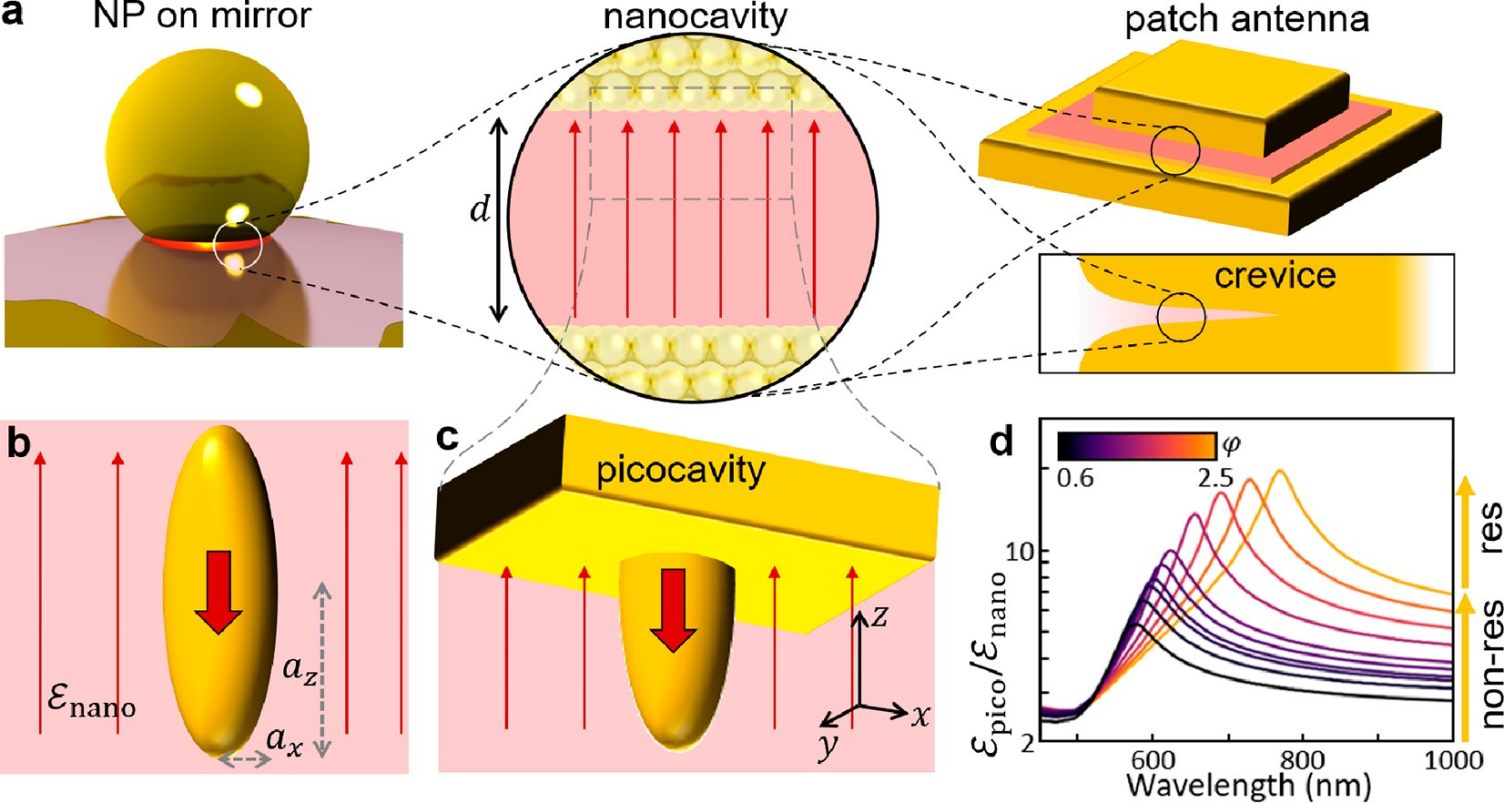
Picocavity analytic polarizability. (a) Plasmonic nanogap-confined optical field, in NPoM, patch antenna, or crevice. (b) Schematic metallic ellipsoid in a uniform nanogap field, showing the induced dipole (solid arrow). (c) Half-ellipsoid embedded in metal facet, axes as marked. (d) Analytic field enhancement at the sharp tip vs aspect ratio φ for a Au picocavity, showing broadband (nonresonant lightning rod) and resonant contributions. (Adapted with permission from ref (6). Copyright 2021 American Chemical Society.)

Surprisingly, the ability to confine visible light to such scales had not previously been considered feasible, even though it is likely a frequent phenomenon. Nanoscale crevices in typical gold or silver jewelry harbor these modes, but to efficiently couple in light of one-thousand-fold larger free-space wavelength requires more sophisticated nanoscale structuring. Many plasmonic geometries such as metal nanorods or near-field tips provide field concentration. A consistent architecture that has proved a useful workhorse for picocavities is the nanoparticle-on-mirror (NPoM). This conveniently combines an antenna with a metal–insulator–metal (MIM) nanothick waveguide between a nanoparticle facet and a metal mirror (Figure 1a). The MIM spacing is set by a dielectric molecular (or crystalline) layer which scaffolds the gap, while the nanoparticle image dimer from the mirror provides the antenna resonance that couples efficiently to free space photons. However, picocavities may occur in any geometry where the internal optical field is large enough to move single atoms out of the facets.

## Simple Model of Picocavities

In this section, a simple analytic model for picocavities is developed that can be widely used to evaluate different concepts. This model of the picocavity field in the nanogap matches full theories and simulations reasonably well and is based on an atom-size metallic ellipsoid in a quasi-uniform field *E*_nano_ inside the gap (Figure 1b). This arises from each nanogap plasmonic mode inside the MIM (Figure 1a), where for small gaps of *d* < 10 nm the perpendicular field (*E*_*z*_) in the gap dominates. The normalized polarizability of the metallic ellipsoid in a dipole approximation is given by
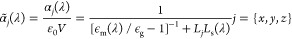
1where *V* is the volume of the half-ellipsoid with semiaxes *a*_*j*_, ϵ_m_ is the metal’s permittivity (typically Au), and ϵ_g_ is the permittivity of the gap medium (e.g., molecular monolayer),^2^ with *z* perpendicular to the metallic facet (Figure 1). *L*_*j*_ are structure parameters accounting for the polarization anisotropy of the ellipsoid with ∑_*j*=1_^3^*L*_*j*_ = 1. The dominant polarizability here is α_*z*_, for which *L*_*z*_ depends simply on aspect ratio φ = *a*_*z*_/*a*_*x*,*y*_ (see eq S1).^3^ To account for half-embedding the elliptical asperity in metal (Figure 1c) using image charges (valid for atomic-scale structures), we include the multiplicative structure factor^4^
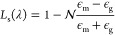
2where  = 0.19 (see SI).

This picocavity plasmon combines a nonresonant “lightning rod” part with a similar-sized resonant part (Figure 1d). Resonances occur when the denominator of eq 1 is zero, and using a simple Drude model for the plasmonic metal, ϵ_m_ = ε_∞_ – λ^2^/λ_p_^2^ leads to
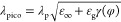
3where
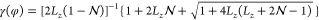


**Figure 2:**
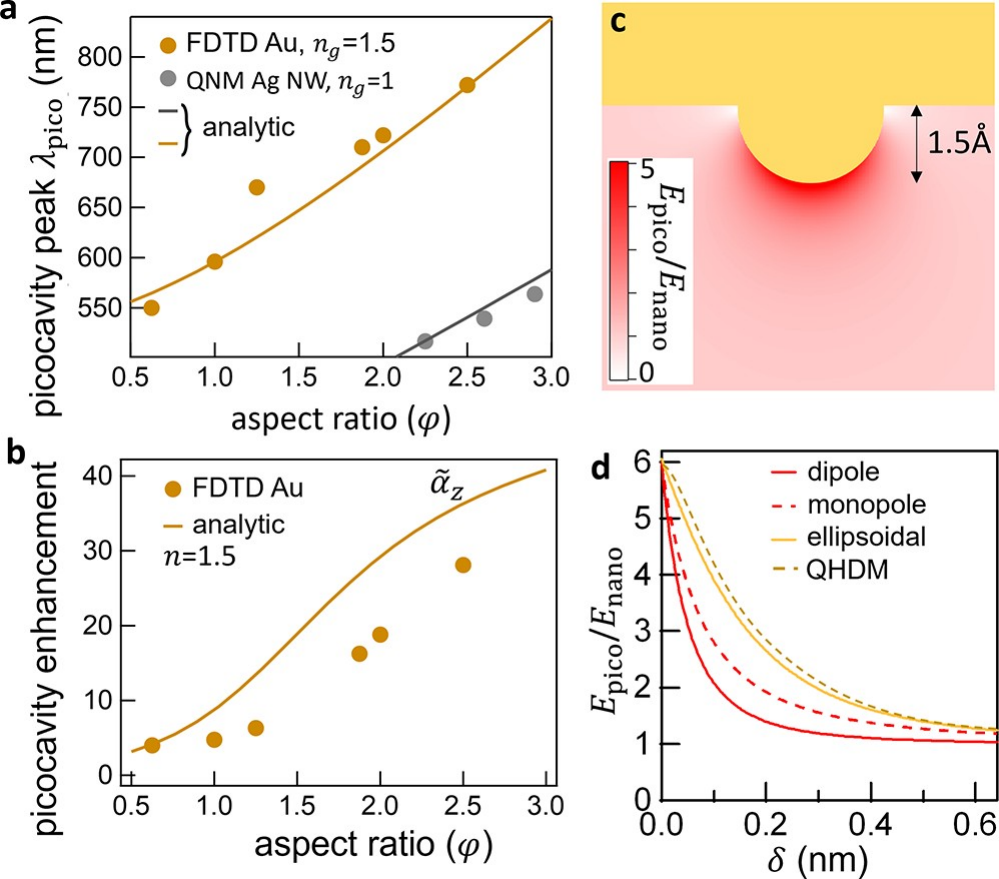
Picocavity spectral tuning and spatial field. (a) Comparison of hemiellipsoid picocavity resonant wavelength model (lines using eq 3) with FDTD calculations for gold^6^ and quasi-normal mode calculations for silver nanowires.^9^ (b) Picocavity field enhancement at the tip for increasing aspect ratio φ of the hemiellipsoid using FDTD and in an analytic model (line using eq 1) vs FDTD calculations for gold.^6^ (c) Dipolar field distribution outside Au hemisphere (0.3 nm diameter). (d) Spatial decay of near field below the picocavity for distance δ outside the Au atom radius, comparing dipolar spherical, dipolar ellipsoidal, and monopolar decay with near-field from the quantum hydrodynamics model.^11^

This analytic expression for λ_pico_ matches full theories well (Figure 2a). Maximum field enhancements at the picocavity tip (on resonance) from eq 1 are EF_pico_ = |α̃_*z*_(λ_pico_)|, set by the imaginary part of the denominator evaluated at λ_pico_ (Figure 2b). Given nanocavity enhancements EF_nano_= 300–500 in NPoM structures,^5^ the total field strength in picocavity hot-spots easily exceeds EF_tot_ = EF_nano_·EF_pico_ > 1000.

Fields around the picocavity have been calculated in many approximations including finite-difference time-domain simulations (FDTD),^6^ quantum time-dependent DFT,^7,8^ quasi-normal mode (QNM) solutions,^9^ finite-element methods (FEM),^10^ and quantum hydrodynamic models (QHDM)^11^ among others. In all, the field resembles our dipolar model (eq 1).^8^ For the spherical picocavity (φ = 1, for φ ≠ 1 see SI), the absence of field parallel to the metal surface sets the central dipole ***p*** = α_*z*_*E*_nano_*ẑ*, with the outside picocavity field *E*_pico_(*r̂*) = *E*_nano_ + [(***p.r̂***)***r̂*** – ***p***]/(4πϵ_0_*r*^3^) or
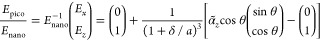
4where *r* = *a* + δ (the distance outside the metal atom of radius *a*) and *E*_nano_*ẑ* is the quasi-uniform nanocavity field in the gap (Figure 2c). We note that *E*_nano_(λ) has its own resonant spectrum (see below). The decay length of this enhanced field away from the adatom surface is 0.7 Å for a dipolar field distribution (and 1.1 Å if monopolar, Figure 2d), while the on-resonance decay length estimated using quantum hydrodynamics for a cone of base 1.2 nm and height of 0.3 nm is 2 Å (Figure 2d). Although electron spill-out and Landau damping have some contribution (QHDM, Figure 2d), the ellipsoidal dipolar model (SI) captures the decay length of 1.6 Å reasonably well for this flattened cone.

From this field profile, the effective mode volume *Ṽ* of the picocavity can be estimated using
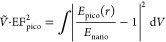
where we take the integral over the gap half-space (Figure 2c) and exclude the original nanocavity field. This can be simply evaluated using eq 4 in the spherical case to yield
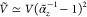
which gives *Ṽ* ≃ π*a*^3^ < 0.01 nm^3^ [see SI §2 in ref (9)] matching estimates near spherical nanoparticles^12^ shrunk to single atoms. With *a* = 0.15 nm for Au, this classical estimate is too small since full QNM simulations^9^ give 0.25 nm^3^, and spill-out effects (Figure 2d) can increase it further. The field inside the metal thus contributes significantly to the mode volume. The ultimate limit to confinement for visible light remains to be clearly theoretically quantified but is on the order of 0.1 nm^3^.

**Figure 3:**
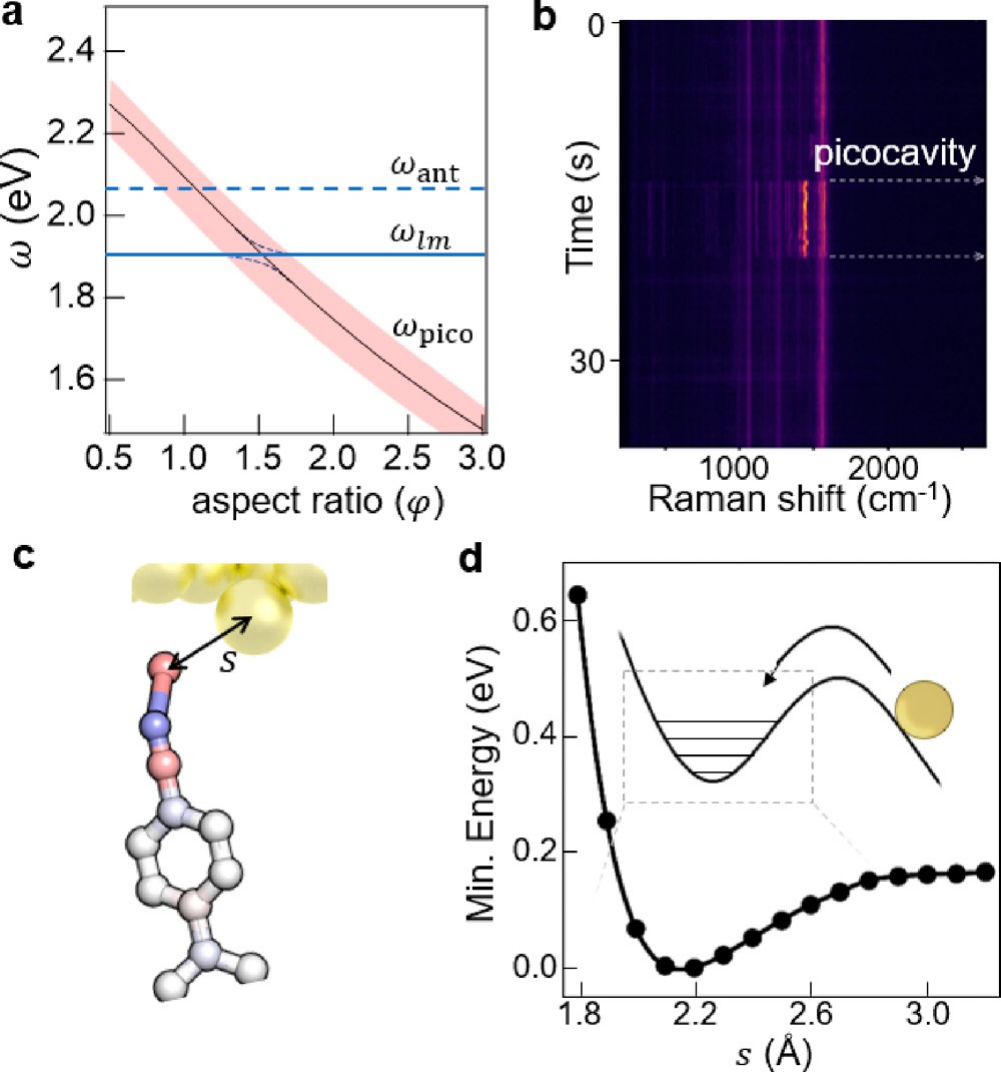
Picocavity, nanocavity, and antenna mode tuning. (a) Picocavity energy tunes with aspect ratio φ, crossing the nanocavity and antenna plasmons, to give efficient free-space coupling when all are degenerate. (b) SERS spectra of the NC-BPT monolayer in the NPoM gap, showing new vibrational lines from a single bond, appear when a picocavity forms. (c) Coordination bond forming between a Au adatom and the tip of the nearest molecule. (d) Calculated DFT of a Au adatom in the vicinity of the NC-BPT molecule, showing a metastable state at 2.2 Å N–Au separation. Reprinted with permission from ref (18) © The Author. Only a few Au vibrational states are thermally excited in this coordination bond at room temperature (inset).

Given this model for picocavities of different metals, aspect ratios, and gap materials, the coupling to nanocavity plasmon modes needs to be quantified. Electromagnetic models show that when the picocavity mode *E*_pico_ tunes through the nanocavity mode *E*_nano_ (which is little affected by the adatom as it occupies a negligible fraction of the mode volume), they anticross to give mixed states *E*_eff_^±^(*r*) = 1/2{*E*_nano_(*r*) ± *E*_pico_(*r*)}, which are split in energy (Figure 3a).^9,11^ The effective mode volumes of these mixed states at their anticrossing point^9,11^ are given by *Ṽ*_eff_^–1^ = 1/2(*Ṽ*_pico_^–1^ + *Ṽ*_nano_^–1^). Since the nanocavity volume is so much larger, the mode volume is then *Ṽ*_eff_ = 2*Ṽ*_pico_ ≃ *V* (the volume of the ellipsoid).

The energy splitting Δ*U* between the *E*_eff_^±^ states arises from their coupling strength, which is controlled by the normalized overlap integral^13^
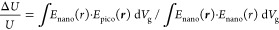


This integral can be evaluated for hemispherical picocavities to give
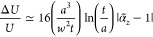
5for a nanogap thickness *t* and facet diameter *w*. For the parameters used in ref (9) which give 0.1 eV splitting at 2.1 eV, using *w* = 7 nm, *a* = 0.2 nm, and *t* = 1 nm in eq 5 yields a 4% splitting in good agreement. For single atom picocavities in NPoM experiments, using *w* = 20 nm, *a* = 0.15 nm, *t* = 1 nm, and φ = 1.0–1.3 (from ref (6)), we obtain splittings < 1%. Comparing with typical picocavity line widths (Figure 1d) which exceed 10% of the mode frequency, such splittings will not be spectrally resolved (Figure 3a). The mixing is however important for efficiently coupling light into the picocavity, while ensuring that the mode volume remains small. Picocavity-induced perturbations to nanocavity scattering spectra will thus be only a few percent, requiring sensitive experiments to detect them.^14^ While desirable to construct larger atomic-scale structures in nanocavities, these are constrained by extremely strong surface forces (see below), and only single (or few) atom picocavities are observed. Recent papers proposing to develop (ultra)strong coupling with single emitter electronic transitions using picocavity fields^15−17^ are intriguing but must be treated with caution currently (see SI).

Two resonance conditions must be simultaneously satisfied to observe such tightly confined picocavity modes from free space. The resonant nanocavity modes^5^ (ω_lm_) must be intense at the spatial location of the picocavity and be close in energy (Figure 3a). To also efficiently couple light from free space into the nanocavities requires the antenna mode frequency ω_ant_ of whichever nanostructure is used (NPoM, MIM, patch antenna, etc., tuning mainly set by height)^5^ to be near-resonant with the same nanocavity modes (ω_lm_).

## How to Observe Picocavities

Picocavities are seen so far through the enhanced SERS of a molecule in the immediate vicinity of an adatom (Figure 3b). The power dependence of anti-Stokes to Stokes Raman emission quantifies large optomechanical coupling strengths, giving vibrational pumping^1^ even at low CW powers (<100 μW) and quadratic power-scaling of anti-Stokes emission.^19^ Picocavity SERS of single molecules is up to 10-fold stronger than combined SERS from all other (100–500) molecules giving nanocavity SERS. This is because gold adatoms do not just passively enhance near-field light but form “coordination bonds” with atoms at the molecule end. Extensive DFT calculations show that for each molecule there is a stable adatom-molecule position, which in simple cases well matches picocavity SERS data. For instance, in cyanobiphenyl-4-thiol (NC-BPT) monolayers giving R–C≡N–Au picocavities, the N–Au coordination bond extracts 0.3e^–^ from the C≡N bond, which thus weakens from 2242 to 2175 cm^–1^ at a stable separation of 2.2 Å (Figure 3c,d).^18^

Variations in the adatom position produce a wandering of picocavity SERS emission, alongside completely stable nanocavity lines. If enough picocavity SERS lines from the same adatom are observed simultaneously, correlating spectral positions allows full reconstruction of the configuration of the single molecule at the metal surface.^18^ This promises real-time ambient observations of catalysis, molecular electronics, surface chemistry, electrochemistry, and sensing (see below). It is thus vital to gain a full understanding and corroboration of the picocavity geometry, chemistry, and pico-optics. Unfortunately, while surface metal adatoms are well-known from STM and electron microscopy, observing them in situ under ambient conditions under optical illumination has proved harder.

## What is Known about Picocavities

To prove picocavity SERS phenomena come from single metal adatoms, coordination-bonded to single molecules, several pieces of evidence are important:

(A) Measured picocavity formation energies^1^ match adatoms (∼1 eV) for both Ag and Au (extracting more atoms requires more energy).

(B) Adatom symmetry breaking alters the Raman selection rules, as observed^1^ (since picocavity optical fields significantly vary along a single bond, Figure 2b,d).

(C) Adatom-molecule coordination bonds seen in SERS are transient and fluctuate in time.

(D) Adatoms only amplify the SERS of a single neighboring molecule, as observed from its vibrational wandering in time (though occasionally two molecules with correlated wandering are seen).

(E) Simulations show only single atom features can reduce the optical field volume below 1 nm^3^ as experimentally measured.

Theory shows that light is localized to mode volumes < 1 nm^3^, perfectly consistent with Maxwell’s equations in a quantum description. Given this, it is puzzling why picocavities are not seen initially, when laser irradiation starts. Both upper and lower Au facets of the MIM are not typically single-crystal or defect-free, but picocavities are never observed without light irradiation (and only then, for intensities above a threshold). Since typical ultraflat Au mirrors used have atomic steps every 5–10 nm, this is surprising. The only conclusion is that reconstruction must take place whenever AuNPs bind to a molecular layer on the mirror. Estimating the van der Waals (VdW) force^20^*F*_VdW_ = *Aa*_NP_/3*d*^2^ for Au Hamaker constant *A* ∼ 1 eV suggests for *d* = 1 nm gaps and *a*_NP_ = 40 nm NPs that *F*_VdW_ ∼ 3 nN over a 20 nm wide facet. This corresponds to 10 MPa (100 atm) ∼ 1 pN/Au atom, or 40 meV per close-packed molecule in a self-assembled monolayer (SAM) nanogap spacer (typically attaching to 11% of Au sites). While SAM Youngs moduli are 0.1–1 GPa, metal facets are ductile enough to rearrange under this pressure, giving single-crystal facets no matter what their initial state.

Picocavities are subsequently created by light (though potentials can also be used^21^). Such optical forces are puzzling since the fields (Figure 2) at these laser powers give optical tweezer forces ∝∇*E*_pico_^2^ of ∼1pN, while pulling the adatom out by Δ*z* = 0.3 nm costing Δ*U* = 1 eV requires *F* ∼ Δ*U*/Δ*z* ∼ 1 nN.^22^ This thousand-fold greater force demands new theory. Experiments show a universal power dependence to the picocavity generation rate that scales with the static polarizability of the atom at the molecule tip.^22^ The optical forces thus involve not the molecular refractive index but its quantum-mechanical polarizability. Several observations are important:^22^

(F) Adatom generation is probabilistic not deterministic.

(G) Adatom decay is also induced by light, at similar rates to formation (adatoms are stable in the dark).

(H) Adatoms are harder to generate at lower temperatures.

(I) Adatom formation rates saturate at higher laser power.

Alternatives rejected include:

(I) hot-atom effects where a 2 eV plasmon is deposited at a single surface atom to kick it out (analogous to hot electrons but should not depend on lattice temperature);

(II) optical forces which tilt the surface potential to drive out surface atoms but would prevent them from returning, instead exponentially inducing roughening;

(III) light-induced Au atom quantum tunnelling through the surface barrier has negligible probability;

(IV) melting of NPs is excluded by measuring their temperature from anti-Stokes/Stokes ratios of nanocavity SERS lines, typically heated by ∼10 K.

The sole explanation identified is that light decreases the barrier for adatom formation. This happens through light-induced VdW attraction between the molecule tip atom in the most intense light and the weakest-bound Au surface atom. The picocavity field (Figure 2b) polarizes the electron cloud around the molecule tip, which induces free electron currents in the metal surface. Solving self-consistently gives strongly enhanced attractive forces.^22^ We emphasize theory breakthroughs are required for integrating photons into DFT calculations,^23^ not yet realistically feasible. However, this model does explain all observations noted above and why picocavity formation rates depend on molecule, laser power, laser wavelength, gap size, facet (*hkl*) plane,^24^ facet metal (Au, Ag, Pd), and temperature. Further experiments with multiple laser wavelengths that identify where on each facet picocavities are pulled out agree with this mechanism.^6^ Such experiments also suggest that φ = 1.0–1.3 (*cf*. Figure 2), consistent with the predicted adatom site on the (111) surface. They also imply reduced adatom barriers near facet edges, expected since the coordination number is lower there.

We note that picocavities do not only form in nanocavities (likely even existing on jewelry); however, optical forces must be strong enough. In nanocavities, these are enhanced by EF_nano_^2^ ∼ 10^5^, so light intensities ∼ 1 W/μm^2^ are required outside nanocavities.

## Picocavity Effects in Applications

**Figure 4:**
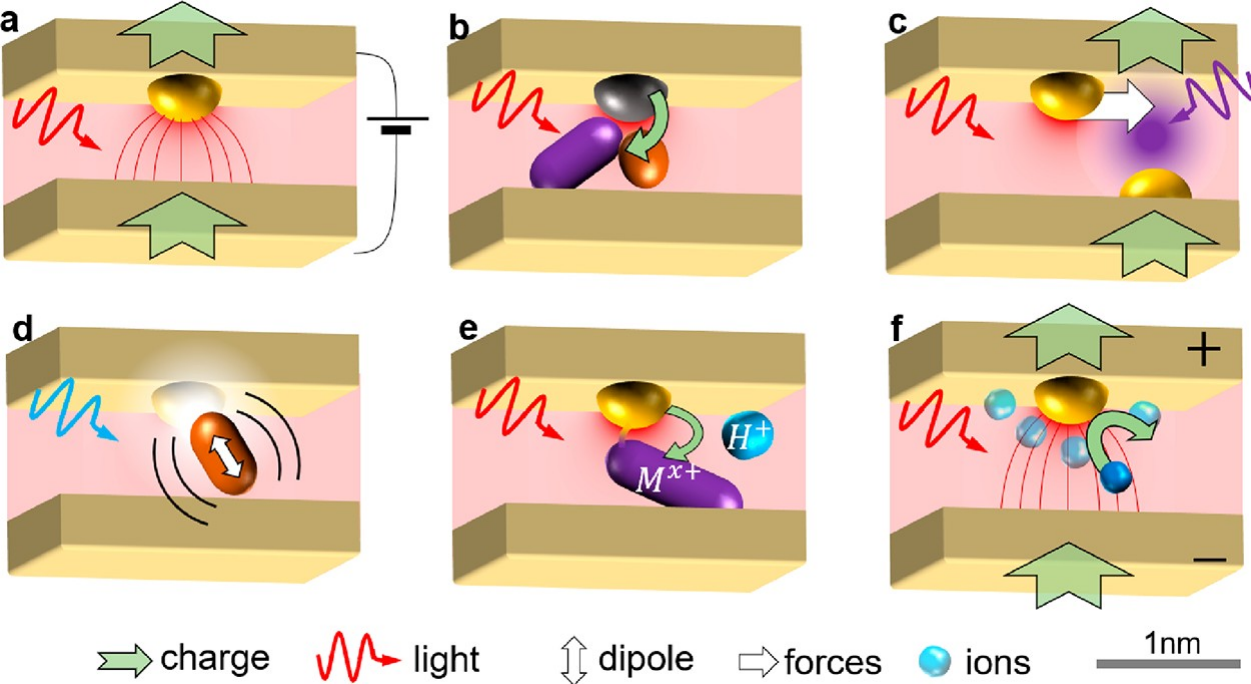
**Picocavity-influenced devices**. (a) Electron tunnelling at asymmetries. (b) Catalytic reactions. (c) Light-driven lateral atomic switching of electrical conduction. (d) Light emission and strong coupling. (e) Light-induced redox chemistry. (f) Surface electrochemistry and ion shell structures.

A number of implications follow from the optical (or electrical) creation of picocavities:

(i) **Quantum tunnelling devices** (Figure 4a): Connecting symmetrical molecules across electrically contacted nanogaps reveals asymmetry in zero-bias photocurrents. DC currents can flow either way, slowly vary in time and with irradiation, randomly directed in each device. Picocavities explain the geometrical rectification seen in recent experiments,^21^ from tunnelling conductance driven by 50 mV “optical” bias (>*k*_B_*T*/*e*) at 100 μW illumination. This suggests molecular (opto)electronic devices which can clock charge through at 100 THz optical frequencies.

(ii) **In-situ single-site photocatalysis** (Figure 4b) can track at the single-molecule level how reactants and products favorably combine at single metal adatoms. Being able to watch the influence of coordination bonds and how this triggers photoreactions greatly aids a nanoscale understanding of catalytic mechanisms.^25^

(iii) **Pico-tweezers** (Figure 4c): Light-enhanced VdW forces open the way to all-optical atomic force microscopies, using multiple colors to excite and laterally translate individual atoms or sheets. Moving adatoms changes electrical paths, giving optical memristive elements.^26^ Single-atom switching delivers low-energy IT devices that can be controlled under ambient conditions.

(iv) **Quantum optical devices** (Figure 4d) are in prospect based on addressing single atoms, bonds, electrons, or molecules in the picocavity field. Only a few quantum states are thermally excited in the picocavity potential formed by a Au–N coordination bond, with fast optical addressing capable of driving entangled states. Single spins and charges are accessible through spin–orbit coupling.^27^ Electronic dipoles of dye molecules or semiconductors (perovskites, TMDs, etc.) coupling to picocavities may give extreme Purcell factors with ultrastrong coupling or pico-lasing but break present light-matter formalisms.

(v) **Pico-chemistry** (Figure 4e) gives access to alternative optical-driven reactions. The extreme opto-mechanical coupling reported^1^ (exceeding room temperature) selectively injects energy into molecules without allowing thermalization, exciting up vibrational ladders. Stimulated Raman scattering selects particular bonds, going beyond coherent control techniques to sculpt reaction coordinates. Despite the confined gap, diffusive access to reactants and products has already been proven. Picocavities also influence vibrational strong-coupling, potentially explaining adatom catalytic reactivities.

(vi) **Single-molecule electrochemical processes** (Figure 4f) can be tracked in real time at picocavities. Single-molecule redox has been observed to influence picocavity formation and decay,^28^ and even single (de)protonation events can be tracked in real time to reveal the local electrochemical and pH landscape.^29^ Complex questions tackled include the solvation of ions at metal surfaces, organization of water and solvents, and light-induced electrocatalysis.

(vii) **Optically controlled hot-spot sensing** through the light-induced VdW interactions can sift through trace molecules and optically attract those of highest tip polarizability. This suggests unusual light-controlled chromatography, where binding-unbinding rates (and hence elution rates) are influenced by light intensity and color.

## Remaining Challenges for Picocavity Science

Many fundamental questions remain. One is the role of charge transfer. While DFT claims adatom coordination bonds are partly ionic, it is unclear how lifting Au adatoms off a surface can locally polarize the surrounding free charges and what oxidation state all atoms are in. This is especially pertinent for the thiol bond typically used to anchor molecules on the lower Au mirror. It is believed that thiol binding plucks Au atoms out of the substrate as Au^(I)^ “staples” (so not observed as picocavities).^30^ The influence of this partially charged mixed S/Au^(I)^/vacancy atomic layer has unknown influence on the optics of metallic Au (but will not affect bonds on the other end, e.g. C≡N–Au). Picocavities do form on the lower mirror,^31^ suggesting that picocavity-staple geometries give similarly strong coordination bonds. Experiments using nonthiol binding systems such as Ru[bpy]_3_ give similar picocavities. It thus appears that the molecular tips play a crucial role in picocavity formation and decay rates. Generally, redox-active molecules give a profusion of picocavities unless chemically stabilized,^28,32^ which likely reflects interactions of picocavities with electron transport and transfer.

The influence of self-assembled monolayer molecular packing is similarly not known, nor how this rearranges to allow Au adatoms to penetrate the molecular layer. In the (stable) NC-BPT system, every picocavity has a different vibrational spectrum, despite the unique energetic minimum from DFT calculations. Formation times of picocavities are <50 μs^33^ but record only when the adatom is within 3 Å, thus amplifying SERS 100-fold. On the other hand, picocavities show two decay rates;^22^ the fast rate matches picocavity formation rates, while the slower one may arise from atomic reorganization of the vacated surface pit that prevents adatoms dropping back in (without many surface rearrangements). Picocavities might suppress their subsequent local creation, although analysis suggests not.^22^

Note, fluctuations from picocavities are important in *every* SERS measurement (although ignored), because their dynamics leads to broadened lines hiding a profusion of different processes, especially when employing long integration times. Picocavity generation clearly resculpts facets but is suppressed for adatoms of higher energy, for instance using (100) facets on nanocubes.^24^

Different dynamics control the wandering of picocavity SERS lines of ∼4 cm^–1^/s. The optically measured adatom diffusion rate *D* ∼ 0.5 Å^2^/s can be used (assuming constrained damped Brownian motion) with the coordination-bond potential (spring constant *k* ∼ 5 eV/Å^2^) to extract a relaxation time τ = *k*_B_*T*/(*kD*) ∼ 50 ms, which is extremely slow compared to formation times or vibrational frequencies.^18^ Changes in pH or redox landscape occur on the same time scale. This suggests that Au adatoms couple to a frictional viscous reservoir, possibly through intermolecular interactions in the SAM, surface waves on the metal facets, or screening electrons in the metal. It is also unclear how Landau damping of electrons can be applied to picocavities.^11^

A challenge for the theory community is to develop rigorous models. Despite observing <100 cm^–1^ picocavity vibrations delocalized over the entire molecule, picocavity fields reach laterally only <0.2 nm into the molecule (Figure 2d). Picocavity SERS intensities and correlations cannot yet be matched by DFT theories and are so far overlooked. Nanogap optical fields of GV/m can be reached (EF_nano_ > 300), capable of breaking bonds and field ionizing. A confluence of different effects must thus be fully connected in picocavities. From deep understanding will come remarkable progress in this successor field to nano-optics.

## Supporting Information Available

Definition of ellipsoidal structure parameters and ellipsoidal dipolar field on-axis (PDF)
