# Supplementary material for: Picocavities: a Primer
Source: Nano Lett. 2022 Jul 6;22(14):5859–65. doi: 10.1021/acs.nanolett.2c01695 (PMC9335881; doi:10.1021/acs.nanolett.2c01695)
Supplement: Supplementary file 1 — nl2c01695_si_001.pdf [file nl2c01695_si_001.pdf]

# Supplementary information

# Picocavities: a primer

Jeremy J. Baumberg[1*]

[1] *Nanophotonics Centre, Cavendish Laboratory, University of Cambridge, Cambridge CB3 0HE, UK.*

**Definition of ellipsoidal structure parameters**

The relevant structure parameter needed is

$$L_z = \begin{cases} e^{-3}(1-e^2)(\tanh^{-1} e - e), & e^2 = 1-\varphi^{-2} \quad \text{if } a_z > a_{x,y} \\ e^{-3}(1+e^2)(e - \tan^{-1} e)\,, & e^2 = \varphi^2 - 1 \quad \text{if } a_z < a_{x,y} \end{cases} \tag{S1}$$

where $a_j$ are the semi-axes of the ellipsoid, and $\varphi = a_z/a_{x,y}$.

**Ellipsoidal dipolar field on-axis**

The dipolar field in the $z$-direction below an ellipsoidal sphere is given by[34]

$$\frac{E_z}{E_g} = 1 + a\left[\frac{1}{2}\ln\left(\frac{\cosh u + 1}{\cosh u - 1}\right) - \left(\cosh u - \frac{1}{\cosh u}\right)^{-1}\right]$$

where $\cosh u = (1+\delta/R_z)/\sqrt{1-\varphi^{-2}}$ .

**Comparison of analytic to full theory**

We note the constant $\mathcal{N}$=0.19 is tuned to match the analytical results with numerical simulations. The agreement of our analytic model with full theory is still impressive given its simplicity, though we note it has not yet been directly measured (despite being the basis of TERS). The over-estimate of the maximum field by 30-50% likely comes from different treatments of rounding geometry at the atomic tip.

**Prospects for picocavity strong coupling**

We note also that while estimates of the Purcell factors in a picocavity can exceed $10^6$, these come with strong effects of quenched emission [ref 9]. A number of recent papers enthusiastically propose to develop strong (or ultrastrong) coupling with a single emitter electronic transition using the enhanced local fields of picocavities [15-17]. We caution that such theories need to be comprehensively developed including all the effects of metal electrons on the neighbouring electronic dipole, in their relaxation, vibronic coupling, as well as Purcell-enhanced emission. This is a fascinating but yet unexplored area, which preliminary results show are confusing.
